# Lived Experiences of Patients Suffering from Acute Old World Cutaneous Leishmaniasis: A Qualitative Content Analysis Study from Iran

**Published:** 2018-06-13

**Authors:** Alireza Khatami, Maria Emmelin, Rezvan Talaee, Akram Miramin-Mohammadi, Nessa Aghazadeh, Alireza Firooz, Berndt Stenberg

**Affiliations:** 1 Center for Research and Training in Skin Diseases and Leprosy, Tehran University of Medical Sciences, Tehran, Iran; 2 Department of Public Health and Clinical Medicine/Epidemiology and Global Health, Umeå University, Umeå, Sweden; 3 Department of Clinical Sciences, Social Medicine and Global Health, Lund University, Malmö, Sweden; 4 Department of Dermatology, Kashan University of Medical Sciences, Kashan, Iran; 5 Department of Dermatology, Tehran University of Medical Sciences, Tehran, Iran; 6 Department of Public Health and Clinical Medicine/Dermatology and Venereology, Umeå University, Umeå, Sweden

**Keywords:** Cutaneous leishmaniasis, Qualitative research, Quality of life

## Abstract

**Background:**

The aim of this study was to explore the experiences of patients who suffer from acute cutaneous leishmaniasis in Iran, focusing on quality of life.

**Methods:**

The study was conducted at two different sites in Iran in 2010–2011. Individual in-depth interviews were conducted with six men and six women parasitologically confirmed acute cutaneous leishmaniasis. Interviews were recorded, transcribed verbatim, and translated into English. Qualitative content analysis was used for data analysis.

**Results:**

The participants, aged 23 to 63yr, had mild to severe disease. Based on the analysis four main themes were developed. “Fearing an agonizing disease” reflects patients’ experiences of disease development resulting in sadness and depression, “struggling to cope” and “taking on the blame” both illustrate how patients experience living with the disease, which included both felt and enacted stigma as major social concerns. “Longing for being seen and heard” refers to patients’ experiences with healthcare as well as their expectations and demands from communities and healthcare to be involved in closing the knowledge and awareness gap.

**Conclusion:**

Mental and social dimensions of cutaneous leishmaniasis were complex and adversely affected patients’ lives by causing psychological burden and limiting their social interactions. Health authorities have to plan programs to increase the disease awareness to prevent the existing stigma to improve patients’ social condition and medical care.

## Introduction

Leishmaniasis is a neglected tropical disease (1). Leishmaniases are a group of diseases caused by different species of the intracellular protozoan, *Leishmania* (2). The parasite is transmitted by the bite of infected sand flies. The disease is endemic in 98 countries, mostly developing ones. About 350 million individuals are at risk of getting different types of leishmaniasis worldwide, and 1.5–2 million new cases of leishmaniasis occur each year, of which 75% are cutaneous (1–4). The majority of cutaneous leishmaniasis (CL) cases occur in ten countries: Afghanistan, Algeria, Brazil, Colombia, Costa Rica, Ethiopia, Iran, Peru, Sudan and Syria (1).

Iran is endemic for CL and almost all CL cases in Iran are caused by either *L. tropica* or *L. major* and in 2012, the Iranian Ministry of Health (MoH) registered 16597 and 4350 cases, caused by *L. major* and *L. tropica*, respectively (4). However, the real number of CL cases could be as high as 100000 cases per year (2).

From a clinical perspective, almost all acute Old World CL (AOWCL) cases in Iran can be divided into two forms: anthroponotic cutaneous leishmaniasis (ACL) and zoonotic cutaneous leishmaniasis (ZCL). ACL is also known as dry, urban or late ulcerative form and is generally attributed to *L. tropica.* ZCL which is caused by *L .major* is also known as wet, rural, or early ulcerative form CL. In human, the initial sign of infection is the appearance of an erythematous papule or nodule at the feeding site of the female sand fly. It appears within one week to three months after sand fly bite. In a typical ZCL infection, the primary lesion usually develops into an ulcer with a violaceous border, which heals spontaneously after several weeks or months, resulting in a scar. Ulceration is not a characteristic feature of ACL lesions. Due to presence of a thick adherent scale on the lesion, hyperkeratosis is the dominant feature of ACL lesions (5–7). Many treatment modalities have been used in the treatment of CL, but pentavalent antimony including meglumine antimoniate (MA) are considered as the first line drugs for treatment so far. They have to be administered only as intramuscular, intravenous or intralesional injections and could be associated with severe side effects and significant discomfort (8). There is lack of strong evidence regarding effective, safe and inexpensive treatments for AOWCL (8, 9). There is no vaccine available for prevention of CL for general human use (10).

The psychosocial impact of CL has been studied in a few quantitative papers. In Turkey, patients with CL had significantly higher levels of anxiety and depression and were less satisfied with their body image as compared to controls (11). CL patients with active lesions also had a more reduced quality of life (QoL) in comparison with patients with healed scars (11). Using Dermatology Quality of Life Index (DLQI) in Kerman, Iran, CL had a moderate to very high effect on the QoL of about 40% of patients (12). The most affected domain was “symptoms and feelings” (12). Using DLQI and SF-36, “Symptoms and feelings” was also detected as the main affected domain in another study from Iran (13). In Kabul, Afghanistan, based on focus group with women, CL implies being socially excluded from communal life and illustrated how CL can cause both social and emotional trauma (14). However, to the best of our knowledge, there are no published qualitative papers focusing on capturing individual experiences of living with CL in Iran.

The aim of this study was to explore perceptions and experiences among patients living with CL as a basis for developing a disease-specific questionnaire focusing on quality of life in CL patients.

## Materials and Methods

### Study setting

The study was conducted at two different sites in Iran: Center for Research and Training in Skin Diseases and Leprosy (CRTSDL), Tehran University of Medical Sciences located in Tehran capital of Iran, which is a referral center for CL patients in Iran with about 140CL patients per year referred from all over Iran (15), and Golabchi Clinic in Kashan about 210 kilometer south of Tehran, where CL is endemic (16). The aim was to represent experiences from patients living in CL endemic (Kashan) and non-endemic (Tehran) areas of Iran.

### Study design

We used a qualitative study designed based on individual in-depth interviews since we were interested in the lived experiences of patients with the diagnosis of CL. Qualitative content analysis to capture the manifest and latent meaning of their experiences were used as a framework for analyzing the data (17).

### Sampling of informants

Informants were sampled purposively to reach maximum variation in regards to the severity of their disease. All informants were parasitologically confirmed CL case. In Tehran, direct smear, culture on NNN (Novy-McNeal-Nicolle), medium, and polymerase chain reaction (PCR) were used for CL diagnosis confirmation. Direct smear was the used technique in Kashan for parasitological confirmation. According to expert opinions and for practical reasons, CL severity was categorized in three levels: mild, moderate and severe. Patients with more than 5 lesions, lesions larger than 5mm in the largest diameter of induration, ulcerative lesions on the face, and sporotrichoid lesions were classified as “severe” cases. Patients with only one or 2 lesions with the largest diameter less than 30mm on locations other than face and ears were considered as “mild” cases and patients within between lesion number and size were defined as cases with “moderate” disease.

We aimed to capture experiences from both men and women. Healthcare professionals at the two clinics (in Tehran and in Kashan) asked patients that fulfilled the above criteria if they were interested to participate in the study. If so they were contacted and informed about the aim of the study to settle on time and place for the interviews.

### Data collection

Data collection took place from Oct 2010 to Nov 2011. All interviews were conducted face-to-face at the respective clinics in rooms that allowed for privacy. All interviews were digitally recorded and followed a thematic guide including three main content areas: “disease development”, “living with the disease”, and “healthcare and community”. During and after the interviews reflective notes were taken by the interviewer to facilitate elaboration on important issues in the forthcoming interviews. The interviews took between 40–75min.

In total, twelve interviews were held with three men and three women recruited at CRTSDL and three men and three women at Golabchi Clinic. These interviews were regarded sufficient to capture the range of experiences and in the last interviews, not much new information emerged.

### Analysis

All interviews were transcribed verbatim into Persian and later translated into English to facilitate joint analysis within the research group. The analysis followed the procedures suggested by Graneheim and Lundman (17). This implied condensing the identified meaning units prior to coding the text. Codes were later clustered and categories and sub-categories were developed to capture the manifest meaning of the text. Finally, themes were constructed to interpret the more latent meaning. During the analysis process, the interpretation was continuously discussed in the research group. An example of the process of analysis from condensed meanings to codes, subcategories, and categories is presented in Table 1.

**Table 1.** An example of analysis process from condensed meaning units to categories

**Condensed meaning unit**	**Code**	**Subcategory**	**Category**
**"I became sad when I got CL. Probability of remaining scars is annoying"**	Becoming sad, fearing remaining scars	Becoming sad	Sensing responsibility
**"When I see people avoiding me because of my CL, or when they think it is communicable, I feel sad."**	People avoid me, think it is communicable, making me sad		
**"My lesion’s appearance is awful. I dislike it"**	Disliking own appearance	Reacting with self-disgust	
**"I became angry after I got CL"**	Becoming angry	Feeling angry	
**"I regret that I did not use a bed net and I got CL"**	Regretting not using bed-net	Regretting own acts	

### Ethical considerations

The study protocol and its attachments were reviewed by the Ethical Committee of Research in Medical Sciences of CRTSDL, Tehran University of Medical Sciences, Tehran, Iran, and the study received clearance on Dec 1, 2007.

Prior to starting the interviews, the informants were asked for written consent and for permission to record. The interviewer gave the informed consent form to each eligible participant prior to the interviews. The informed consent form clearly indicated how confidentiality would be kept and that participation was voluntary and would not influence the medical treatment. The whole session would be recorded. Participants were asked to read the form carefully and were provided with further clarifications if needed before signing the form.

If the patients needed treatment, they were treated free of charge. The study was conducted in accordance with the Declaration of Helsinki.

## Results

Twelve patients were from 23 to 63yr old and all had got CL in the endemic areas. Those who lived in Tehran got it during travels to endemic areas and those who got in Kashan were living in Kashan or its suburbs. Numbers of male and female participants were equal. Four cases had mild CL, another four had moderate CL, and the rest of them suffered severe CL (Table 2).

**Table 2.** Demographic and clinical characteristics of the interviewees

**#**	**Sex**	**Age (Years)**	**Occupation**	**City of Residence (Where CL was got)**	**CL Severity**
**T1**	Female	48	Teacher	Tehran (Mashhad)	Moderate
**T2**	Female	45	Housewife	Tehran (Damghan)	Severe
**T3**	Male	27	Caretaker	Tehran (Damghan)	Severe
**T4**	Male	33	Bookbinder	Tehran (Zavareh)	Mild
**T5**	Male	34	Aircraft fueler	Tehran (Shiraz)	Moderate
**T6**	Female	38	Teacher	Tehran (Zavareh)	Mild
**K1**	Female	31	University student	Kashan (Kashan)	Moderate
**K2**	Male	50	Well excavator	Ravand (Ravand)	Moderate
**K3**	Male	63	Businessman	Ravand (Ravand)	Severe
**K4**	Male	40	Scrap dealer	Ravand (Ravand)	Mild
**K5**	Female	40	Housewife	Kashan (Ravand)	Mild
**K6**	Female	23	University student	Kashan (Kashan)	Severe

The analysis of the interview texts resulted in four main themes: “Fearing an agonizing disease”, “Taking on the blame”, “Struggling to cope”, and “Longing for being seen and heard” (Table 3).

**Table 3.** Summary of main finding indicating themes, categories, and sub-categories

**Content area**	**Category**	**Theme**
**Disease development**	Understanding the disease gradually Perceiving early skin lesions Considering other skin diseases	Fearing an agonizing disease
	Feeling disease’s mental impact Starting to worry about consequences Being physically and socially limited	
**Living with the disease**	Sensing responsibility Regretting own acts Feeling angry Reacting with self-disgust Becoming sad	Taking on the blame
	Facing stigma Being rejected	
	Feeling isolated Finding themselves excluded	
	Preventing others’ reactions Hiding disease or self Avoiding close contact	Struggling to cope
	Internalizing reactions from others Understanding others reactions Tolerating others behavior Appreciating others’ support	
**Healthcare and community**	Finding treatment difficult but acceptable Feeling the need for medical care Playing little role in treatment schemes Being satisfied with treatment results	Longing for being seen and heard
	Noting limited knowledge on CL Meeting unawareness in the community Experiencing ignorance within the healthcare system	
	Urging for prevention High treatment costs Considering current preventive methods as unsatisfactory	

The themes reflect four content areas elaborated in the interviews. The first covers patients’ experiences of disease development while the second and third themes illustrate how the patients experience living with CL. The last theme refers to patients’ experiences with the healthcare system, their expectations, and their demands. The results are presented under the headings of the themes with categories as sub-headings and subcategories incorporated in the text in italics. Examples of quotations from the interviews are used to illustrate how the interpretation is linked to the data. The quotes are labeled with T1-6 and K1-6 for patients from Teheran and Kashan, respectively.

### Fearing an agonizing disease

This theme captures patients’ worries when they first experience symptoms and how these are perceived as scary and threatening. The supporting categories show how some of the clinical characteristics and the course of the CL lesions affect the patient’s mental status.

### Understanding the disease gradually

The patients perceived early CL lesions to resemble many other skin disorders, for example, simple insect bites, boils, or allergic reactions.

However, CL did not follow the usual courses of an insect bite or other aforementioned diseases, which were expected to heal within a few days. Patients’ confusion became more evident when the lesions grew instead of healing. They considered other skin diseases and started to worry. In particular, for those who were not familiar with the disease, this enlargement, and progression of the lesions, associated with development of relatively big, red, wounded, sometimes painful skin lesions, was worrying. Symptoms and signs of CL could be quite perplexing as described below:

“… It was itching and red, then it became larger, like the size of a pea. It was itching and burning. After two months it became larger and still was itching. Then it became purulent. I tried to drain the purulent material myself several times, but each time the wound appears again and become purulent.” T3.

### Feeling the disease’s mental impact

Feeling mental trauma because of CL was due to different experiences. Some patients were afraid because they misdiagnosed their own disease with more serious disease, commonly those known to be contagious, stigmatizing diseases and notorious for being difficult to manage:

“I thought I might have shaken hand with someone who has had AIDS or something. Or maybe that person’s hand was bleeding and I had a wound on my hand too…so, I might get it [AIDS].” K4.

These experiences together with misdiagnosis by healthcare providers made the patients fearing an agonizing disease. A patient said that a general physician made a diagnosis of a mosquito bite and prescribed him some antihistaminic tablets and an anti-pruritic ointment, which were not effective. Patients’ experiences of worrying about different aspects of the disease including its course and its outcome contributed to finding CL a disease, which causes mental trauma and preoccupation. Interestingly, in some patients, a confirmed diagnosis of CL was relieving to some extent, although the worrying about long-term outcomes remained:

“I was anxious for myself because of uncertainty about the condition [I got] and what will happen to it.… I was relieved after CL diagnosis was made because of knowing that it was curable. However, I was worrying about the remaining [CL] scar”. T1

This patient showed how starting to worry about CL consequences could make CL a disease, which adversely affected patients’ mental health. Other factors such as having limited knowledge about spreading and transmission routes of CL also contributed to this mental impact.

They limited knowledge of patients about their own disease was associated with distress and having enough information about their CL would facilitate understanding their disease:

“It is better for a person who knows [about CL]. If he/she knows about CL he/she would not be worried. The person who does not know it feels under pressure… A patient who knows about CL could cope better with the disease.” K4. The patients experienced CL as a being socially and physically limiting. Some patients experienced physical limitations which were mainly related to the interfering nature of the CL lesions with their daily work:

“Since my job is basically done using my hands, an elbow [CL] lesion has adverse effects on my performance” T4, or:

“My work is dealing with metal scraps. When I got CL, I could not work for about one month. I had to get a worker to do the job for me…I could not touch the industrial waste [metal scraps]”. K4.

Social limitations were more related to becoming isolated, rejected, and even stigmatized which is also described under the theme “struggling to cope”. Being physically and socially limited contributed to CL being associated with mental impact.

### Taking on the blame

“Taking on the blame” is one of two themes that illustrate how CL patients experienced living with their disease. It represents the internal aspects of living with CL.

### Sensing responsibility

*Regretting own acts* for getting CL showed how some CL patients acknowledged their own role in getting the disease and sensing their responsibility:

“I regret that I did not use a bed net and I got CL” T2.

Some CL patients *felt anger*, which might adversely affect their relations with co-workers and family members. For example, a man with severe CL recalled:

“After I got CL I became irritable and moody. On one occasion, I became really angry at my workplace when someone hit my elbow for fun. Moreover, at home, there was one occasion that my wife asked me to help her for preparing the bed but I told her that I would not help. I became so angry, that I went to another room for sleeping and told her that my hand is painful and I could not even pick up a 1-kg weight. …Anger was because of getting CL.” T3.

Becoming angry was related to sensing responsibility because the patient felt responsible for doing something he was expected to do or avoiding something that he was expected to do as a result of his anger. Reacting with self-disgust and becoming sad were other sub-categories contributing to sensing responsibility.

“I am really sad because I find my CL lesions disgusting. Their appearance is very bad and they are really awful. I always say prayers that it [CL] be completely cured as fast as possible.” T1.

### Facing stigma

Some prominent experiences in CL patients were feelings of being rejected, isolated, and excluded. These experiences showed both enacted and felt stigmatization:

“…Everyone is scared of CL. Like in the mosque that I used to go, there was a woman who had CL where everybody could see her lesions. Nobody would sit beside her or shake hands with her. People pointed her out to each other and talked about her. … She was lonely. … When they stood in lines for prayers (Namaz), people stood away from her at a distance…When I got CL, I experienced these matters myself.” K6, or:

“In the bank, once they saw my [CL] wound, they looked away [dropped their head]. Or in the bakery when I touched the bread to give it to someone, they threw the bread away. It is because of infection [CL].” K4.

In some cases, the feelings were so overwhelming that the patients felt being stigmatized and used the exact word of stigma or being stigmatized to describe their situation:

“I feel being stigmatized after getting CL. I think this stigmatization is imposed by close friends as well as distant ones [local shopkeeper].” T1.

### Struggling to cope

This theme also reflects patients’ experiences of living with CL. However, while the theme “taking on the blame” basically refers to patients’ own role, “struggling to cope” illustrates how CL patients’ react to other people reactions, one of the less explored aspects of the CL patients. CL patients’ interactions with others were spread over a wide spectrum of somewhat contradictory experiences illustrated by two main strategies used to handle others reactions towards their disease.

### Preventing others’ reactions

The first strategy was to prevent reactions from other people to happen, basically through *hiding the lesion or self*. An example of this preventive mechanism was evident from these quotations:

“Even if my brother comes to our home I wear a long-sleeved dress, so they [he] cannot see this part of my elbow.” T1, or:

“Even you may want to hide.” T4.

For some patients, it was quite upsetting and discouraging to constantly feel forced to inform about their disease. This even resulted in telling lies to avoid more distress:

“I did not fear [others reactions], but I was upset that I was being asked about it [CL] all the time, like every 5 to 10min, from my university to home I should have explained it for 20 people. That bothered me. I did not like to answer. In the beginning, I told them it was CL and then I observed that everyone, from young to old, run away from me. They did not take a sandwich or a glass of water from me. The problem was that I had to lie to them to make myself feel more comfortable. I would say it was wart and people stopped asking question when they thought it was wart. Lying to people bothered me. But it was a white lie, it was better than my suffering [for being asked about my lesions too frequently]”.K6.

### Internalizing reactions from others

The second strategy was to see the disease through the eyes of others by indicating that they understood others’ reactions towards them. Some preferred to use the term “tolerating” instead of “understanding”, indicating that while they could not understand or find a rationale for others’ reactions, they tried to accept the situation they were in:

“I thank God that I am really tolerant and I do not struggle with people. I tell my colleagues that I have CL and ask them to hit [as a sign of friendship] on my healthy arm instead of the affected one.” T3.

At the same time patients experienced others’ support:

“In my case, they [my colleagues] gave me my injections at the workplace, although if they did not, I would go somewhere to receive them. They also gave me some leave. … I told them that I recovered, but they told me that I have not recovered yet. They told me that I follow up my disease and if I need something for example money, I phone them. …”.

### Longing for being seen and heard

The last theme reflects CL patients’ encounters with the healthcare system. CL patients experienced being ignored, and excluded from shared decision making for treatment plans, and not receiving proper acknowledgement for their own treatment demands. It is also related to the patients’ experiences in the communities, in which people have limited knowledge about CL or despite having enough information on the disease, they do not behave in accordance with what they know.

### Finding treatment difficult but acceptable

CL treatment was not easy because of its associated side effects. However, when they felt they would benefit they tended to accept the treatment offered.

“I feared of [MA] injections. Once, I received incomplete treatment because of [injection] pain, but later on I completed the [treatment] course. CL treatment course is shorter than the courses for some other health problems. I feel happy about the results of the treatment of CL.” T3, or:

Feeling really uncomfortable also contributed to needing medical care because of the severity of the disease

“When I became uncomfortable because of the pain and itch on my CL lesion, I decided to go to a doctor… I knew that CL would finally heal [spontaneously], but I decided to seek medical advice on its treatment…” T5.

Some patients were prepared to tolerate quite painful complications in order to quicken the healing phase.

“When I received [MA] injections, I felt some malaise … I felt that [MA] injections caused problems for my kidneys because I had to wake up during the nights and go to WC. But in general, the complications were not serious. I have prescribed 3 vials [of MA] each day for 2 weeks, a total of 42 injections. I received them intramuscularly and my [gluteal] muscle became very stiff, so I could not use the last 3 vials. I knew that [MA] injections would heal CL lesions in about 2 wk. I also knew that without treatment CL might take about 6 months to be healed.” T4.

Others felt that they did not play enough roles in making decision about the treatment of their disease and were not satisfied with the physician’s approach to treatment. They had wanted to be more involved even if they were satisfied with the treatment results.

“I have to raise one point; my physician postponed my treatment for 10 d because he did not want to treat me with [MA] injections at first. He told me that the injections would be very painful and I might not be able to walk for some time after I received the [MA] injections, but when I received the injections, they were nothing extraordinary. I think that if he started my treatment earlier, my disease would not become so bad. I imagine patients do not actively participate in choosing the treatment methods. They play too little role in the treatment plan.” T2.

### Noting limited knowledge on CL

The participants described how they met unawareness in the community and clearly saw how this was one of the important factors increasing their suffering:

“…I am a sportswoman and I [have to] wear clothes that are comfortable [which do not cover all parts of the body], and since many people do not know what it [CL] is and whether it is communicable or not, they ask questions or avoid me, and I have to wear certain clothes and also I will remain isolated”. K1.

However, it was not only in the community that the participants had met ignorance about the disease. Even within the health care system, they experienced ignorance and suspicion resulting in them being treated as if the disease was highly transmissible.

“Even once I went somewhere to receive my injection and the nurse who wanted to do the injection feared so much that she refused to inject the medicine… She thought that it is highly transmissible and feared a lot….” T4.

Some had concrete suggestions of how to increase community awareness and underscored that the information given needs to be repeated regularly to really make a difference.

“… Maybe not enough information [in the community]. They give people brochures about CL every 10yr, maybe. If these brochures and handouts are given to people every 2–3 months, once a year and everyone knows about CL and gets better education, it would become much better.” K6.

Others emphasized the importance to adjust the information provided to different groups of people and to write in an understandable language.

“I would like to see more public education about CL. There must be some public areas where people gather and discuss different aspects of the disease [CL]. The society is full of people of different kinds, young, old, educated, illiterate, etc. The information should be easily understandable by everyone. Not just a printed hand-out that they give to people.” K1.

### Urging for prevention

The participants also emphasized the need for prevention from different perspectives. For those who were less economically privileged the urge for prevention was based on the high treatment costs:

“The disease [CL] has to be prevented. You have to go after treatment, spend money for it. Have to look until you find a clue. When I was first bitten by CL [mosquito], I could not figure it out. I got unemployed. I spent money. …I had to pay for transportation fees.” K2.

However, the need for prevention was not raised only by the poor. Moreover, patients from households with moderate to high incomes not only acknowledged the importance of prevention but considered current preventive methods as unsatisfactory:

“I think CL prevention is very important. I hope a vaccine be developed for CL soon. Current preventive measures like using insect repellent sprays and even using bed nets are not completely effective”. T4.

## Discussion

Exploring the lived experiences of patients suffering from acute AOWCL indicated that the disease affects different aspects of the lives of the patients. While most physical problems of CL lesions were quite expectable, the mental and social dimensions of the disease were affected much more than what it was thought. However, we noticed some important experiences by the patients which were related to the course of the disease development and management. The adverse impacts of CL on mental health started with facing an agonizing and fearing disease and continued with worrying, anxiety, sadness, and depression. The main societal problem associated with CL was stigma. These problems together with insufficient information about CL in general population and sometimes among healthcare providers resulted in the patients’ struggle of coping with their situation and longing for being heard and seen by both ordinary people and health authorities. Disseminating knowledge about CL in community and better training of healthcare professional could help to improve CL patients’ conditions.

CL was not a minor health problem (14). While it is not uncommon for other dermatologic diseases to be misdiagnosed by the patients and non-dermatologist physicians, CL misdiagnosis is quite agonizing for the patients because its clinical course is dramatically different from what they expect. For example, a patient whose early CL lesion was misdiagnosed with an ordinary insect bite and received treatment for the latter, worried when he/she observed that instead of healing, his/her lesion grew much larger and became purulent. According to our experience, patients might mistake CL with a wide spectrum of dermatological diseases from boils to skin tumors, leprosy and HIV/AIDS infection. These misinterpretations of symptoms and signs were particularly evident when the patients had no previous information about CL. This association between knowing about CL and better QoL was also reported, patients without previous knowledge about CL has worse QoL in comparison with those who had previous knowledge about it (13).

Depending on the location and severity of CL, it could result in physical limitations for the patients, which could consequently affect their income, because they were not able to do their jobs. However, the impact of unemployment and health expenditure in AOWCL is much smaller in comparison with some more chronic tropical diseases such as lymphatic filariasis (18).

Lack of knowledge about CL among general population was a contributing factor to the CL-associated stigma. Since Kashan is endemic for CL, it was reasonable to assume that similar to patients with CL, people without CL in Kashan were quite familiar with CL and had a better understanding of CL patients’ situation. However, such understanding was not reflected in their behavior towards the CL patients. This finding was similar to results (14), which indicated that while people in endemic areas for CL like Kabul, Afghanistan had an acceptable knowledge about several aspects of the disease, their behavior towards CL patients was not congruent with their knowledge.

Worrying about their disease and its consequences including scar formation was a dominant finding among our participants and some of them also mentioned that they became sad because they got CL. Our findings were similar to results which showed significantly higher anxiety and depression in CL patients than healthy controls (11). Lower QoL and body image satisfaction in patients with active CL lesions in comparison with healthy individuals or those who had CL scars (11).

An important and less explored aspect of CL is the psychological and social effects on the patient’s lives. Our study contributes with examples of feelings and reactions in relation to themselves or other individuals that could result in psychiatric problems such as depression. Our findings are supported by two studies from Iran’s neighboring countries; Turkey and Afghanistan that found depression and anxiety were more frequent among CL patients (11, 14). Depression and sadness because of the disfigurement caused by filariasis could have greater burden for the patients than its physical consequences (19). This greater burden of psychosocial impact could occur in CL patients as well. Regretting own acts and reacting with self-disgust could contribute not only to sadness and depression but also to low self-esteem and social disconnection (19).

Several definitions of stigma were provided (20), of which three, i.e. enacted, perceived, and internalized stigma were described in patients on another neglected tropical disease, lymphatic filariasis. Our CL patients experienced different types of stigma. Some CL patients, mostly those with moderate or severe disease, were rejected, feeling isolated or finding themselves excluded which could be considered as examples of enacted stigma (19, 20). Among the management strategies for this kind of stigma, the patients used denial by telling lies about their disease and hiding their lesions. When they talked about their experiences, some of our participants said some matters that indicated perceived (felt) stigma, such as being aware of other who had CL and experienced discrimination. They were afraid of being treated like those whom they had heard about. Their management strategies were similar to patients who suffered filariasis including modifying social interactions, isolating self, and hiding the affected body parts (19). We also found examples of internalized (self) stigma in our interviews with CL patient like regretting own acts (i.e. guilt), reacting with self-disgust, and sadness (19). CL patients with internalized stigma used the same management strategies (19) of women with filariasis. Another type of stigma was defined “aesthetic” or “unaesthetic” stigma which is caused by visible marks or visible deformities (21–23). Since it was on CL stigma in Surinam that terminology might be relevant to our study as well (23) although we imagine that it does significant overlap with the three former stigmas we have already mentioned and is not a unique entity.

Since CL is very common in some countries, to reduce the CL-associated stigma some strategies have been developed which could be considered cultural rather than individual (22, 24). One example is according to Desjeux that CL was known as ‘little sister’ in some endemic countries where it was very common and became part of normal life just as a family member (24). Another cultural management strategy to counteract CL stigma was pointed in Turkey, in which the older people were accustomed to the CL scar, and called it “a beauty scar” to increase the tolerability and normalization (22). However, the younger generations did not accept the CL disfigurement and scar and considered it as a life-long stigma (22). Severe CL-associated stigma was reported in Kabul Afghanistan, where the disease was very common and the people were knowledgeable about CL (14). This difference might be explained by lack of culturally-built management strategies regarding CL stigma. Because of some wrong believes, more than half of their studied cases mentioned that they would prevent a CL patient from touching and hugging his/her child, and they would not allow a CL patient prepare food in the home (14). In addition, more than 20% of participant in their study said that a mother with CL should not breastfeed her child. Women with CL lesions or scar would face problem if they would like to marry (14). The CL stigma and its social impact in Afghanistan were larger than what it is in Iran (14).

According to some participants in our study, they tried to accept their situation and acknowledge others’ reactions toward their disease by trying to understand or at least tolerate the others. Even some of patients appreciated others’ supports. Therefore, these mechanisms together with some of the aforementioned stigma management strategies helped the CL patients to continue their struggle to cope. Provided an interesting discussion about the potential roles of morals and contextual morals in de-stigmatization of health problems including depression, which might be used in combating CL-associated stigma when the knowledge about that issue become more clear in the future (25). They explained how stigmatized individuals were not able to meet the obligatory and normative moral criteria that were set in a context of a society by socio-economic, cultural, anthropological and political determinants. Acknowledging the interconnection between those who stigmatize and those who were stigmatized (25) stated that understanding and changing the stigma-determining factors could help to combat stigma through modifying the contextual morals.

In Dominican Republic and Ghana on lymphatic filariasis (19, 26), factors such as religious beliefs and hope provided the basis for better coping with the disease. Due to strong religious beliefs in most Iranians, this factor played a role for those who internalized or integrated others’ reactions.

Determinants of the stigma associated with another neglected tropical disease, leprosy were reported, as visibility of the lesions, cultural and religious beliefs, fears of transmission and public health interventions (27). From the interviews with patients and considering Iran’s cultural context, it would be likely that the same determinants play roles in CL stigma. The association of visibility of the lesions with stigma in skin diseases is quite well known, not only in infectious diseases in developing countries but also in non-infectious diseases such as psoriasis and vitiligo in both developing and developed countries (28, 29). Hotez provided a concise and interesting discussion about the importance of stigma in the burden of disease of neglected tropical diseases including CL, which might be not be reflected in a quantitative indicator of disease burden like disability-adjusted life year (DALY) (30). Small DALYs of CL patients (31), from Iran, was in line with Hotez (30) conclusions and indicated the need for qualitative researchers to better explore the impacts of CL on patients’ lives. A multifaceted approach to target CL itself through increasing the availability of affordable care for CL patients and to educate people about the disease was needed to reduce the social burden and the associated stigma of CL (32), we also imagine that developing and implementing healthcare educational and interactive de-stigmatizing programs could play important roles to improve CL patients’ situation.

Despite the patients knew about the self-limiting nature of CL, they sought medical care form health professionals (14). Some patients found their encounters with healthcare providers were not satisfactory. They were misdiagnosed, they were not sufficiently engaged in the decision-making process of treatment, and even were behaved discriminately against. They were aware about the adverse effects of the standard treatment of and even experienced some of those complications; however, they decided to receive the recommended therapeutic regimen. During the interviews, several patients gave answers indicating that they were aware of traditional methods of CL treatment provided by the traditional herbalists or recommended by their family members or friends, but they preferred to be treated by medical doctors. However, none of them had experienced a traditional therapeutic method.

One of the issues that was surprising in our study was the fact that while, most interviewees were quite knowledgeable about CL risk factors like exposure to sand-fly bites, routes of its transmission, and in some cases about the clinical signs and symptoms, most patients acted similarly to individuals who were not familiar with the disease. The patients’ experiences indicated that in endemic areas such as Kashan and Damghan, the general populations were quite knowledgeable but still acted in a stigmatizing way towards CL patients. The patients attributed their experiences to lack of knowledge in the community, although it might be not be explained only by this factor. Indeed, the CL-associated stigma might be a culprit. Most patients asked for better health education programs to tackle this problem. According to the patients, trained healthcare providers’ knowledge about CL was also insufficient and CL patients asked for better training be provided by the responsible organization and authorities such as MoH to improve the situation.

We found it interesting that almost all interviewees answered an open question about “what else they would like to add to what had already been talked about?”, by emphasizing the lack of information and the existing ignorance about CL in the community. They asked for ways to increase the public awareness with emphasis on the potential role of mass media especially Islamic Republic of Iran Broadcasting (IRIB) Television. They believed that visual programs could improve knowledge about CL in those who could not read newspapers, educational pamphlets, and posters.

The participants asked for more effective preventive methods. Although less socio-economically privileged patients considered high treatment costs as a reason for improved preventive interventions, at least one study conducted in an endemic area in Iran reported that with the exception of educational programs, applying other known preventive methods such as using insect repellants, pesticides, and bed-nets were not cost-effective or cost-benefiting (33). Using the Markov decision analytic computer model, development of a vaccine for CL with only 50% efficacy and as little as 5yr of protection in seven Latin American countries with highest incidences of CL in that region would be cost-effective in comparison with standard treatment regimens (34). However, the treatment cost per patient and the morbidity of New World CL is higher than Old World CL (33, 35). Development of new and more effective preventive methods such as vaccines was also one of the demands of relatively affluent CL patients in our study. Even if our interviewees were aware the getting CL for the second time is very unlikely, they still were looking for more effective prevention methods. CL could be prevented in them, but also would be preventable for those who still had not experienced the disease.

### Trustworthiness

#### Strengths

The first author (AK) is an experienced dermatologist having worked with CL for several years at CRTSDL in Tehran and in endemic areas in Iran. To increase the credibility of the study he made multiple visits to the endemic setting (Golabchi Clinic, in Kashan) to assess the situation, build trust with the clinic staff and to familiarize them with the research project. His prolonged engagement also included spending several weeks in the Kashan during the actual data collection period. However, during the interviews, he made efforts to put his knowledge and experiences of CL as a dermatologist within brackets and allow the informants to tell their stories. The analysis was regularly discussed in debriefing meetings within the research group including interdisciplinary competencies. Taking field notes during his stay documenting the research process facilitated keeping and audit trail to account for the dependability of results.

#### Limitations

Only people sought medical care for their CL lesions were included in the study so individuals who had not been coming for medical treatment were excluded. An expert panel had been consulted and it was agreed that it is unlikely that the experiences between the two groups were considerably different. CL requires medical care, which was taken as an indication that this would also be the situation in Iran (14). However, the transferability of our results is limited by only including patients with acute Old World CL (lesions duration less than 6 months). Those who suffer from other forms of CL, which have longer duration, could be considerably different since they have other clinical courses and outcomes. For consent reasons our study only investigated patients who were older than 18yr and we acknowledge this as a limitation since children are a large and important group at risk of having CL in endemic areas.

## Conclusion

Our study demonstrates how CL interferes with patients’ health, both mentally and socially. To provide a better management and reduce the burden of CL, in particular, its associated stigma, further studies to investigate the QoL of CL patients as well as developing and implementing health education programs in endemic areas are recommended.
